# The Tibetan herbal medicines Padma 28 and Padma Circosan inhibit the formation of advanced glycation endproducts (AGE) and advanced oxidation protein products (AOPP) in vitro

**DOI:** 10.1186/1472-6882-14-287

**Published:** 2014-08-05

**Authors:** Ewa Grzebyk, Agnieszka Piwowar

**Affiliations:** Department of Pharmaceutical Biochemistry, Wroclaw Medical University, Wroclaw, Poland

## Abstract

**Background:**

Advanced glycation endproducts (AGE) and advanced oxidation protein products (AOPP) play a pivotal role in the development of diabetes associated diseases. The herbal medicines Padma 28 and Padma Circosan have shown effectiveness in symptoms of diabetes associated diseases and have antioxidant effects. It is not known whether inhibition of AGE and AOPP formation is a mechanism of their action.

**Method:**

BSA was subjected to glycation or oxidation with or without 70% ethanolic extracts of Padma 28, Padma Circosan or with an active control. AGE and AOPP concentrations were analyzed fluorimetrically or spectrophotometrically respectively and by ELISA.

**Results:**

Compared to the positive control Padma 28, Padma Circosan and the active control significantly reduced AGE levels by 58.6%, 56.7%, and 8.14% (fluorimetry) and by 35.48, 34.19, and 19.68% (ELISA). AOPP were reduced by 57.28/66.78% (30’/60’ incubation), by 67.08/71.99%, and by 81.68/86.54% (spectrophotometry) or by 79.98/86.97%, 79.3/84.3% and 77.07/90.31% (ELISA). All results are significantly different (p < 0.001). No difference was found between the effects of the two preparations.

**Conclusion:**

Both formulas significantly inhibited the formation of AGE and AOPP to a similar extent as the active controls. This suggests a possible role for both Padma preparations in the treatment and prevention of diabetes associated diseases.

## Background

Diabetic vascular late complications (DVLC) are multifactorial diseases that can manifest in different clinical forms. Together with other factors such as inflammatory processes and dietary and genetic causes chronic hyperglycemia and oxidative stress are the two main factors causing metabolic disturbances in diabetes mellitus (DM). They lead to glycation and oxidation of macromolecules and tissues, thereby altering their structure and function. The most known and unfavorable results of hyperglycemia and oxidative stress are the modification of proteins, leading to the formation of advanced glycation end-products (AGE) and advanced oxidation protein products (AOPP). These are being intensively investigated for their pivotal role both in the pathogenesis and development of DVLC. AGE and AOPP formations are important mechanisms among metabolic disturbances occurring in diabetes. AGE and AOPP have a complex structure. They undergo modifications such as crosslink reactions and accumulation and bind to the same receptor, the so called receptor for advanced glycation end products (RAGE), which is the key component of the intracellular signaling pathways leading to further metabolic disorders in diabetes. The processes of glycation and oxidation mutually potentiate and intensify each others deleterious effects and thus constitute a vicious circlecalled glycoxidation. Glycemic control and reduction of risk factors of diabetes and its associated diseases stand at the center of therapeutic strategies. These are based on lifestyle modification programs (weight loss and physical exercise) as well as pharmacological treatment including oral antihyperglycemic agents and insulin [1]. However, intensive glucose lowering showed limited benefits regarding all cause mortality and deaths from cardiovascular causes in a meta-analysis [2].

In view of these data and considering the importance of diabetes-associated diseases, an extended therapy and prevention approach is needed for diabetic patients. Herbal medicines may play a role in various phases of such an integrative diabetes management [3]. On the one hand herbal medicines usually contain numerous antioxidative substances, which may be helpful in the prevention of increased oxidative stress and inflammation as usually occurs in diabetic patients [4–6]. On the other hand some herbal preparations are known to lower the blood glucose level, to inhibit AGE formation, prevent acute myocardial ischemic or to have antibacterial properties [3, 5, 7–9]. In view of the pivotal role diabetic complications play in the disease process and mortality of diabetes such preparations might be highly valuable in treatment and prevention of DM and its complications.

Among herbal preparations with anti-inflammatory and anti-oxidant effects there is the Tibetan herbal multicompound medicine Padma 28. The formula contains 20 plants as well as calcium sulphate and natural camphor and is well-known in Europe since the 19^th^ century [10]. It has been marketed in Switzerland for the last 35 years and is used in circulatory disorders with symptoms such as a tingling sensation, formication, feeling of heaviness and tension in the legs and arms, numbness of the hands and feet and calf cramps. An almost identical formulation is available under the name Padma Circosan (sometimes also Padma Basic), in different European countries. The formulas act according to a multi-target mechanism on different pathogenic factors of atherosclerosis [11].

Clinical data have shown Padma 28 to be effective in micro- and macroangiopathies such as atherosclerotic diseases, e.g. peripheral arterial occlusive disease (PAOD) and angina pectoris, as well as in symptoms of disturbed circulation such as paresthesias, pain and swelling in the extremities and wound healing [12–15]. These symptoms have a special relevance in diabetic patients.

Various experimental studies suggest a multi-target mode of action for Padma 28 and Padma Circosan, which, except the omission of one ingredient (Aconiti tuber), is identical to Padma 28. Among others the anti-oxidative and anti-inflammatory properties are mentioned. Padma 28 was found to be a strong iron chelator and radical scavenger [16]. Furthermore, Padma 28 inhibited lipid peroxidation [17] and protected LDL from oxidation [18].

These data as well as clinical case reports in diabetes caused paresthesias and polyneuropathia [19, 20] support the use of Padma 28 and Padma Circosan in diabetes and diabetes associated diseases. However, nothing is known about their effect on AGE and AOPP formation. The aim of the current study was to examine, whether these herbal preparations are able to inhibit the formation of AGE and AOPP *in vitro* and if so, whether there is a difference between the activities of these two formulas.

## Methods

### Test substances and materials

Padma 28 and Padma Circosan were produced according to good manufacturing practice by Padma Inc. in Switzerland. They were provided as raw powder without excipients and contain calcium sulphate, natural camphor and 20 or 19 herbal drugs respectively. The composition of Padma 28 has been published previously [21].

In this study, 400 mg herbal powder were suspended in 2 ml of 70% ethanol and shaken at 37°C for 30 minutes. The solutions were centrifuged for 15 min at 2200 rpm and the supernatants were filtered through 0.22 um filter (Millipore, USA). Bovine serum albumin (BSA) was used as model protein, which was glycated and oxidated in two individual studies. BSA was used in a concentration of 40 mg/mL, which corresponds to the physiological concentration of albumin in human blood. As a negative control (C-) BSA was dissolved in PBS (pH 7.4), and as a positive control (C+) BSA was incubated with glucose or chloramine as appropriate for the process of glycation and oxidation of proteins. All reagents used in the study were from Sigma (USA). The glucose and chloramine concentrations were chosen based on previous studies. Experiments were performed in different concentrations of extracts and the amount of extracts used was chosen for the optimal readability in the detection methods appliedd. The experiments were performed in three series, each time in triplicate. Each sample was sealed in a separate flask, and all were incubated in Heidolph Polymax 1040 device at 50 rpm in a darkened room.

### Glycation

BSA was incubated with 500 mg/mL glucose for eight weeks at 37°C (positive control C+) or with PBS alone as negative control (C-). For the actual test Padma 28 and Padma Circosan extracts were added 0.8/8 (v/v) and for the active control aminoguanidine (A, 50 mM), a known inhibitor of protein glycation, was used.

Every week, samples of 0.8 mL were collected. After 48 hours of dialysis in PBS with a one-time exchange of solution after 24 hours, the samples were frozen at −80°C for future analysis. The level of advanced glycation end products (AGE) was measured fluorimetry with the modified method described by Munch et al. [22, 23]. Briefly the characteristic fluorescence with excitation at 370 nm was measured at 440 nm with a spectrofluorimeter Perkin-Elmer LS50B. The results were converted into arbitrary fluorescence units (AFU) according to the formula:


where:

F - measured fluorescence intensity of the sample

R - dilution of the sample (R = 500)

V1 - volume of the cuvette in μL (V1 = 1000)

V2 - sample volume in μL (V2 = 20)

To determine the AGE concentration with the immunoenzymatic method the OxiSelect Advanced Glycation End Products (AGE) ELISA test (CELL BIOLABS, INC, San Diego USA) was used. The test was performed according to the manufacturer's instructions, with the use of appended reagents and was read on Stat Fax 2100 spectrometer (Awareness Technology, Inc., USA) at a wavelength of 450 nm and 630 nm differential filters.

### Oxidation

BSA was incubated with chloramine T (20 mM, positive control C+) for 60 minutes at 37°C or with PBS alone (negative control C-). The test substances were added in a final concentration of 0.8/8 (v/v), vitamin C, as a known inhibitor of protein oxidation, was used as an active control (10 mM).

After 30 minutes, a first set of samples of 0.8 mL was collected. After 48 hours of dialysis in PBS, with one exchange of solution after 24 hours, all samples were frozen at -80°C for future analysis.

The level of advanced oxidation protein products (AOPP) was measured to assess the degree of albumin oxidation. The spectrophotometry method described by Witko-Sarsat et al. [24] was applied. The measurement of AOPP is based on their reaction with potassium iodide in the presence of acetic acid. The derivatives were measured spectrophotometrically at 340 nm. For the calculation of the concentration of AOPP a calibration curve was generated using increasing concentrations of chloramine-T solution as reagent for iodination and the following formula was applied:


where:

C - concentration of AOPP in the material

A - absorbance measured at a wavelength of λ = 340 nm

R - coefficient taking account into dilution (R = 0.70922)

B - slope of the calibration curve (B = 0.0141).

To determine AOPP concentration by the immunoenzymatic method the AOPP Kit (Immun Diagnostic, Benshein Germany) was used. The test was performed according to the manufacturer's instructions and was read on the Stat Fax 2100 spectrophotometer at a wavelength of 340 nm and 630 nm differential filters. All experiments were performed first with a blank to assess whether the red color of the extract influenced the test readings. This was only the case in the ELISA measurements of AOPP. These results were corrected accordingly with the readings of the blanks.

All tests were run in duplicate (controls) or triplicate (test substances) and the experiments were each repeated twice, the data are reported as the mean ± SEM.

### Statistical analysis

The results were statistically analyzed using parametric tests. The presented results obtained from the analysis of Student’s t-test in Statistica PL (version 10.0). A p-value of < 0.05 was regarded as statistically significant.

## Results

### Glycation process

**Figure 1:**
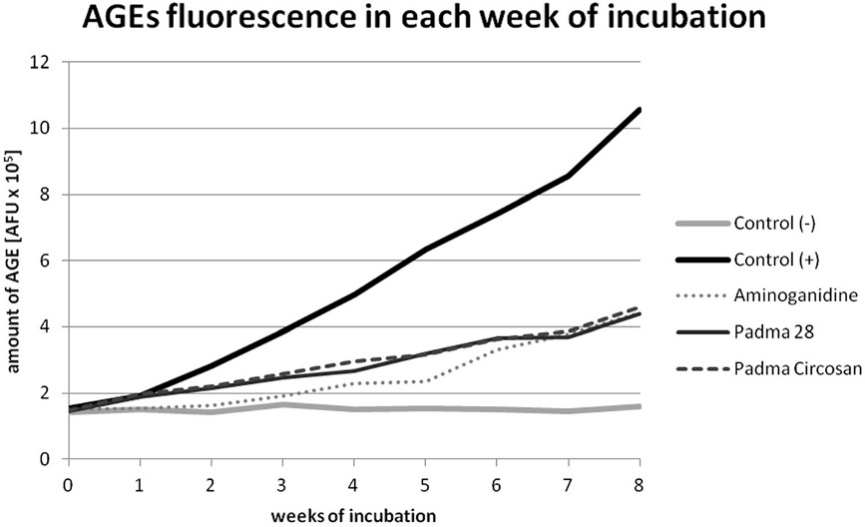
**The inhibition of the formation of AGE by Padma 28, Padma Circosan and the active control aminoguanidine over the 8 weeks of incubation as measured fluorimetrically.**

**Figure 2:**
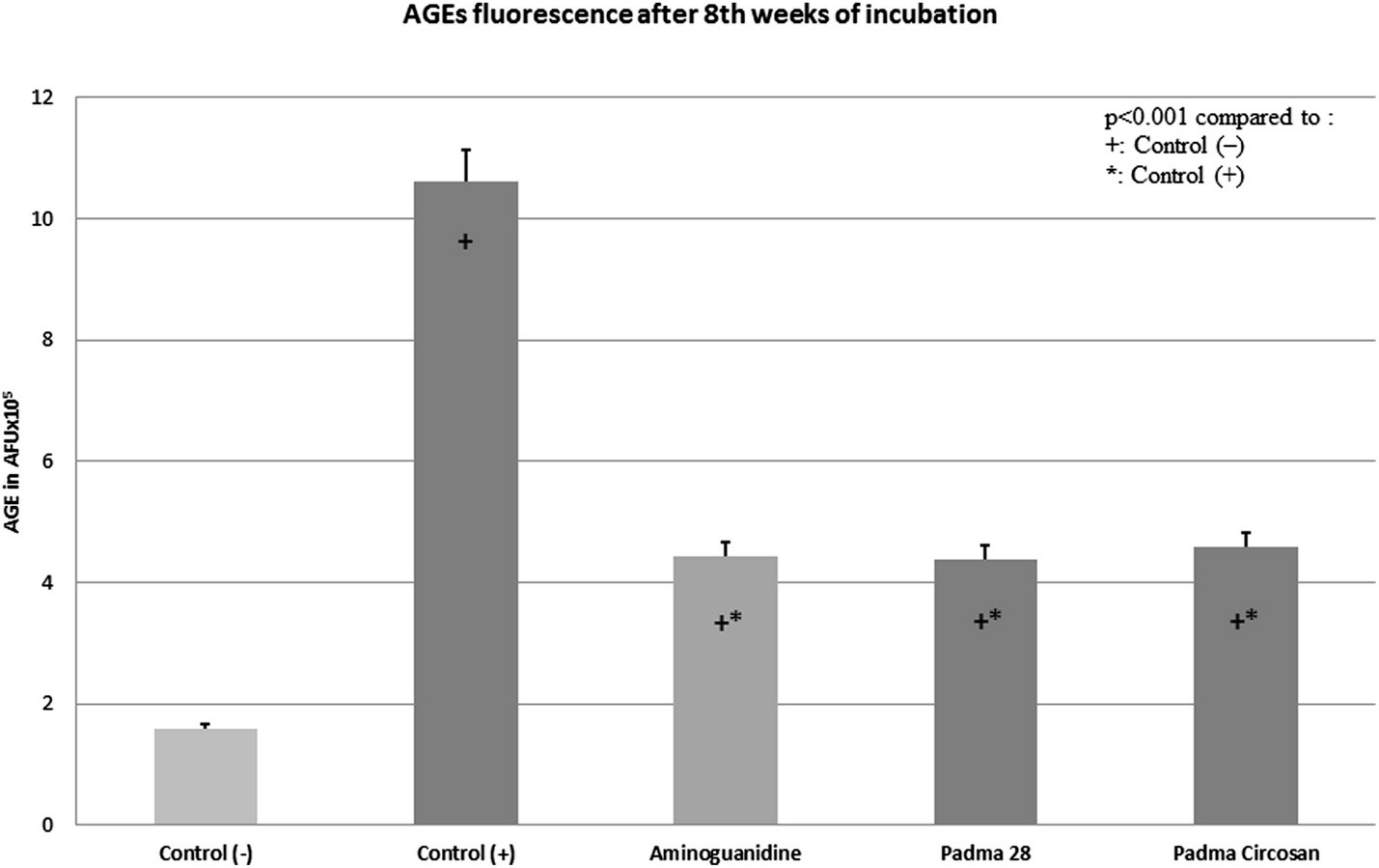
**The concentration of AGE [AFUx10** ^**5**^ **] measured fluorimetrically after 8 weeks of incubation.** Experiments were conducted in three series in triplicate each, data are presented as the mean ± SEM.

**Figure 3:**
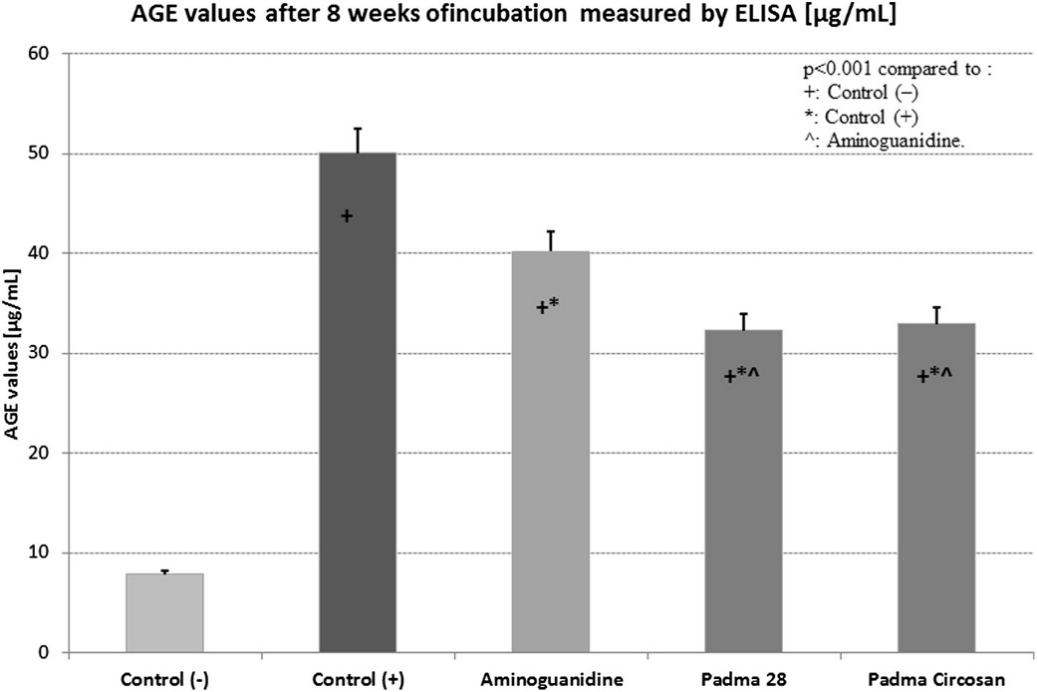
**The concentration of AGE [μg/ml] measured by ELISA after 8 weeks of incubation.**

The ethanolic extracts of both Padma 28 and Padma Circosan as well as the active control inhibited the formation of AGE significantly as measured fluorimetrically as well as by ELISA. In the fluorimetric analysis the level of AGE in the samples of glycated BSA albumin (positive control C+) was significantly higher than non glycated BSA albumin (negative control C-) from week 1 and was 6.69 times higher at week 8 (p < 0.001, Figure 1, Figure 2). All three substances (the active control aminoguanidine, Padma 28 and Padma Circosan) showed an inhibitory effect on protein glycation (significantly from week 2). Both the test substances showed an almost identical level of glycated proteins as the active control. Compared to C + the AGE after 8 weeks of incubation were reduced by 58.14% (aminoguanidine), 58.6% (Padma 28) and 56.688% (Padma Circosan, all p < 0.001, Figure 2).For confirmation of these data AGE concentrations were analyzed also with ELISA and the results expressed as mean ± SEM of all 3 test series (Figure 3). Due to the low sensitivity of the test reliable data could only be obtained at 8 weeks of incubation.

The concentration of AGE in the positive control (C+) was 6.38 times higher than in the negative control (C-, p < 0.001). Compared to C + aminoguanidine, Padma 28 and Padma Circosan showed a significant inhibitory effect on protein glycation and thus lower concentrations of AGE (p < 0.001). While aminoguanidine inhibited AGE formation by 19.68%, Padma 28 by 35.48% and Padma Circosan inhibited it significantly more by 34.19% respectively (both p < 0.001 compared to C + and to A).

No statistically significant difference was found between Padma 28 and Padma Circosan, neither with the fluorimetric method nor with ELISA.

### Oxidation process

**Figure 4:**
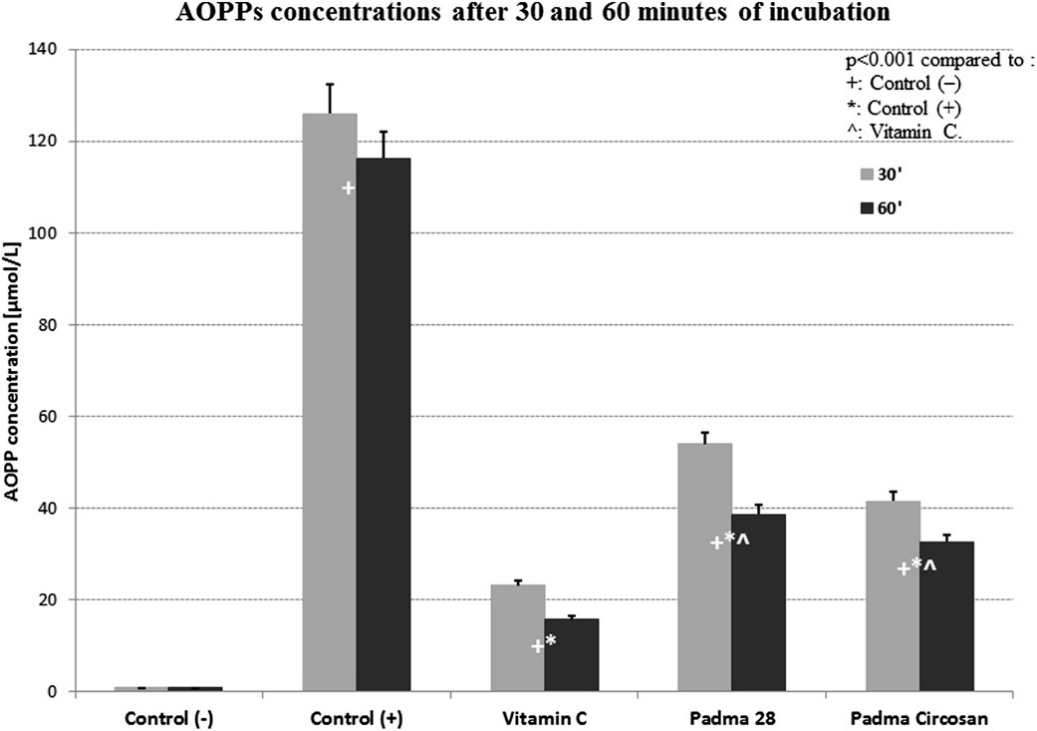
**The concentration of AOPP after 30 and 60 minutes of oxidation measured spectrophotometrically.**

**Figure 5:**
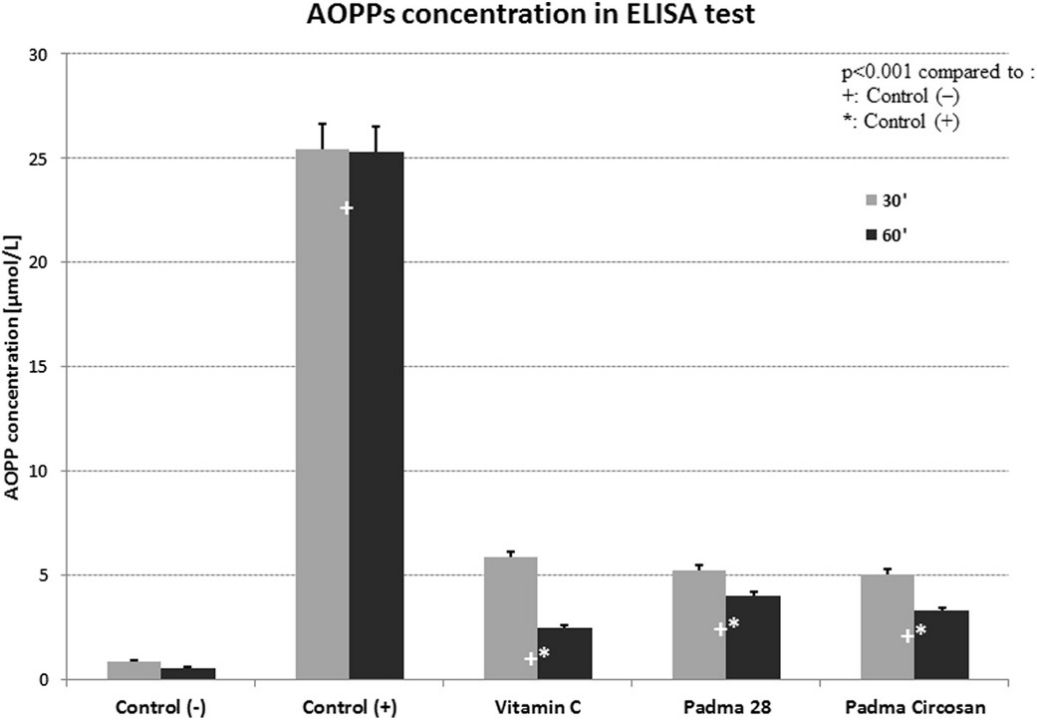
**The concentration of AOPP after 30 and 60 minute of oxidation measured with ELISA.**

Both Padma 28 and Padma Circosan as well as the active control (vitamin C) inhibited the formation of AOPP significantly in the spectrophotometric as well as in the ELISA test (Figures 4 and 5).The concentration of AOPP as analyzed with spectrophotometry was 150.33 and 149.22 times higher in the positive control C+ than in the negative control C- after 30 minutes or 60 minutes of incubation respectively (both p < 0.001). The active control vitamin C as well as the test substances Padma 28 and Padma Circosan showed an inhibition of BSA oxidation by 81.68% and 86.54%, by 57.28% and 66.78%, and by 67.08% and 71.99% after 30 or 60 minutes respectively (Figure 4, all p < 0.001 compared to C+). The difference between vitamin C and the test substances was statistically significant at both incubation times (p < 0.001), but not between Padma 28 and Padma Circosan.These results were confirmed by measurements of AOPP concentrations with the ELISA kit. The results of the BSA oxidation are expressed as means ± SEM of all 3 Series after 30 and 60 minutes of incubation (Figure 5).

The results show that the concentration of AOPP in the positive control is 29.0 and 47.8 times higher than in the negative control after 30 or 60 minutes of incubation respectively (both p < 0.001). Vitamin C, Padma Circosan and Padma 28 reduced the oxidation of BSA by 77.07% and 90.31%, by 79.98% and 86.97%, and by 79.3% and 84.3% after 30 or 60 minutes respectively (all p < 0.001).

No statistically significant differences were found between the active control and the two test substances.

## Discussion

The prevalence of diabetes mellitus type 2 and therewith also of diabetes-associated diseases is growing globally. Nearly 350 million people (about 3,8% of the population) suffer from diabetes and 70% to 80% of diabetic patients eventually die because of diabetes-associated diseases and DVLC, especially cardiovascular disorders [25, 26]. Although these are multifactorial diseases involving among others genetic, dietary, life-style factors, and immunologic pathogenic processes, AGE and AOPP formed as a results of chronic hyperglycemia as well as oxidative stress are pivotal in metabolic disturbances occurring in diabetes. Glycation and oxidation processes also occur at low levels in healthy people and naturally increase with advancing age, but the increase is much steeper in diabetes patients, even if hyperglycemia is well controlled [27]. Accelerated glycoxidative modifications of macromolecules, especially proteins, are a major factor leading to damage of blood vessels and nerves, finally resulting in micro- and macroangiopathies and others diabetes-associated disturbances [3, 27].

AGE are a heterogeneous group of molecules formed from the non-enzymatic reaction between reducing sugars and free amino groups of proteins, lipids or nucleic acids. The first product of this reaction is a Schiff base, which spontaneously rearranges itself into an Amadori product. These initial reactions are reversible depending on the concentration of the reactants. Then a series of subsequent reactions, including dehydrations, oxidation-reduction reactions, fragmentation and other re-arrangements lead to the formation of AGE. One characteristic of AGE is their ability to form covalent crosslink formations between proteins e.g. cellular matrix proteins, basement membrane proteins, and vessel-wall components, a process which alters their structure and function. Another important feature of AGE is their interaction with RAGE receptors, which are presented on the surface of a variety cells and which leads either to their endocytosis and degradation or to cellular activation and pro-oxidant, pro-inflammatory events [22, 27].

AOPP are also a heterogeneous group of molecules originating as a result of the action of free radicals on proteins, especially albumin and lipoproteins. Chloramine, hypochlorous acid- myeloperoxidase-H_2_O_2_-halide system of activated phagocytes are the main agents participating in AOPP formation *in-vivo*. The structure of AOPP shows a large similarity to that of AGE. Moreover, like AGE, AOPP can react with RAGE receptors, accumulate in tissues and act as inflammatory mediators triggering the oxidative stimulation of neutrophils, monocytes and T-lymphocytes, thus leading to their upregulation through a positive-feedback mechanism [28, 29]. Increased AGE and AOPP formation and accumulation in the vascular tissues have been associated with changes in endothelial cells and function. They are the protagonists in the pathogenesis of diabetes associated diseases and contribute to the DVLC such as hypertension, dyslipidemia and low-level inflammation [29, 30]. Thus, prevention of excessive formation of AGE and AOPP seems to be a very important and promising aspect in the management of diabetes and the prevention of diabetes associated diseases.

In our study, using bovine serum albumin (BSA) as a model protein, the antiglycoxidative properties of Padma 28 and Padma Circosan were analyzed. We revealed strong inhibitory effects of both formulas on AOPP as well as on AGE formation. No difference was found between the activities of these two preparations. Interestingly, the anti-glycative effects of Padma 28 and Padma Circosan seem to be of a similar magnitude as that of the active control aminoguanidine. This was confirmed both by the fluorimetric as well as by the ELISA method. The anti-glycative effects of Padma 28 and Padma Circosan as determined by the immunoenzymatic test were even stronger than the effect of aminoguanidine compared to the results obtained fluorimetrically. The difference between the two methods may result from the high specificity of the ELISA assay. The results show that Padma Circosan and Padma 28 inhibit the glycation process to a similar degree and they show only a slightly stronger inhibitory effect than aminoguanidine, indicating a very strong anti-glycative activity of both formulas. The results determined by the spectrometric method show strong inhibitory effects of Padma Circosan and Padma 28 on the oxidation process, thus providing further evidence of their anti-oxidant activity. After 30 min of oxidation Padma Circosan and Padma 28 show a similar level of inhibition on the protein oxidation process as vitamin C, whereas after 60 minutes the inhibitory effect of vitamin C was more pronounced. However, even after 60 minutes of oxidation Padma Circosan and Padma 28 still show a strong potential to counteract the oxidation of proteins. An extrapolation of these results gained in a cell-free *in vitro* setting to the clinical situation is not possible. First clinical data from single case reports have to be supplemented with further studies to determine the clinical relevance of the effects found in these experiments.

Although vitamin C, functioning as an active control in our experiments, has a known strong antioxidative potential and an inhibiting effect on AOPP formation *in vitro*, the results of clinical trials with vitamin C in diabetic complications are equivocal [31]. One reason for this could be that for an effective detoxification of oxidative stress in the organism a well functioning antioxidative network is needed, in which a variety of antioxidant agents of different strengths as well as enzymatic antioxidants and an intrinsic recycling process and the regeneration of the antioxidant capacity of the system work together. The increase of one of these elements alone, e.g. vitamins C or E, cannot uphold the antioxidative capacity of the system. Because they contain many different antioxidative compounds and, as is the case for Padma 28, additionally activate antioxidative signaling pathways, herbal substances are much better suited in this complicated multifactorial events [32, 33].

## Conclusion

In summary, the results clearly show strong anti-glycative and anti-oxidative effects of Padma 28 as well as Padma Circosan, which are of a similar order than the standard active controls. Moreover, the present study shows that both preparations are able to protect albumin from detrimental oxidative and glycative modification. This is evidence of a so far unknown mode of action which, additionally to the symptomatic treatment of circulatory disorder in general, suggests a special benefit in patients with diabetes. The inhibition of AGE and AOPP formation, causative mechanisms in the development of diabetes associated diseases, thus suggest a possible role of Padma 28 and Padma Circosan in an integrative diabetes management, not only as a treatment of specific circulatory symptoms, but also more generally in the prevention of diabetes associated diseases.
